# Ni-Cu and Ni-Co-Modified Fly Ash Zeolite Catalysts for Hydrodeoxygenation of Levulinic Acid to γ-Valerolactone

**DOI:** 10.3390/molecules29010099

**Published:** 2023-12-22

**Authors:** Margarita Popova, Momtchil Dimitrov, Silviya Boycheva, Ivan Dimitrov, Filip Ublekov, Neli Koseva, Genoveva Atanasova, Daniela Karashanova, Ágnes Szegedi

**Affiliations:** 1 Institute of Organic Chemistry with Centre of Phytochemistry, Bulgarian Academy of Sciences, 1113 Sofia, Bulgaria; momtchil.dimitrov@orgchm.bas.bg (M.D.); ivan.dimitrov@orgchm.bas.bg (I.D.); 2 Department of Thermal and Nuclear Power Engineering, Technical University, 1756 Sofia, Bulgaria; sboycheva@tu-sofia.bg; 3 Institute of Polymers, Bulgarian Academy of Sciences, 1113 Sofia, Bulgaria; fublekov@polymer.bas.bg (F.U.); koseva@polymer.bas.bg (N.K.); 4 Institute of General and Inorganic Chemistry, Bulgarian Academy of Sciences, 1113 Sofia, Bulgaria; genoveva@svr.igic.bas.bg; 5 Institute of Optical Materials and Technologies, Bulgarian Academy of Sciences, 1113 Sofia, Bulgaria; dkarashanova@yahoo.com; 6 HUN-REN, Research Centre for Natural Sciences, Institute of Materials and Environmental Chemistry, Magyar Tudósok krt. 2., 1117 Budapest, Hungary

**Keywords:** lignocellulosic biomass, levulinic acid, fly ash zeolite, γ-valerolactone, Co-Ni alloy

## Abstract

Monometallic (Ni, Co, Cu) and bimetallic (Ni-Co, Ni-Cu) 10–20 wt.% metal containing catalysts supported on fly ash zeolite were prepared by post-synthesis impregnation method. The catalysts were characterized by X-ray powder diffraction, N_2_ physisorption, XPS and H_2_-TPR methods. Finely dispersed metal oxides and mixed oxides were detected after the decomposition of the impregnating salt on the relevant zeolite support. Via reduction intermetallic, NiCo and NiCu phases were identified in the bimetallic catalysts. The catalysts were studied in hydrodeoxygenation of lignocellulosic biomass-derived levulinic acid to γ-valerolactone (GVL) in a batch system by water as a solvent. Bimetallic, 10 wt.% Ni, and 10 wt.% Cu or Co containing fly ash zeolite catalysts showed higher catalytic activity than monometallic ones. Their selectivity to GVL reached 70–85% at about 100% conversion. The hydrogenation activity of catalysts was found to be stronger compared to their hydration ability; therefore, the reaction proceeds through formation of 4-hydroxy pentanoic acid as the only intermediate compound.

## 1. Introduction

Lignocellulosic biomass is a promising alternative to fossil fuel sources because of the increased demand for energy and declining supplies [1,2,3]. Another essential aspect in energy production is the prevention of greenhouse gas emissions, as biomass and biomass-derived fuels are considered carbon neutral. The direct combustion of biomass, however, is not energy efficient, due to its high moisture content and low volumetric energy density; therefore, the possibility of its conversion into liquid or gaseous fuels, so-called biofuels, is being intensively investigated [4,5]. Catalysis plays a crucial role in processing biomass into fuels, their precursors or platform molecules [6,7]. An important step of biomass valorization is the hydrolysis of lignocellulose into a mixture of cellulose, hemicellulose and lignin, and further transformations to C5 and C6 monosaccharides [8]. Among platform molecules, which are produced from biomass, levulinic acid (LVA) is one of the most important for the production of valuable chemicals, such as biofuels, solvents, intermediates for the pharmaceutical industry, etc. [9,10,11,12]. LVA is considered a chemical building block because it contains keto and carboxyl groups in its structure, which enables its conversion into a wide range of derivatives. Moreover, it is one of the top value-added sustainable chemical compounds that are produced from abundant renewable resources [13]. The hydrodeoxygenation of LVA to γ-valerolactone (GVL) is an important process because it can be used as a solvent, fuel additive or intermediate in the production of diverse value-added chemicals. The hydrodeoxygenation of LVA to GVL has been performed in gaseous or in liquid media by applying homogeneous or heterogeneous catalysts [14]. Another issue is the energy efficiency of hydrogen supply for the hydrodeoxygenation process through alcohols, formic acid or molecular hydrogen [15,16,17]. Hydrogen can also be produced by the catalytic steam reforming of biomass-derived LVA, which allows the integration of the hydrogen production with the hydrogenation processes [18].

Heterogeneous catalytic processes offer advantages compared to homogeneous ones, such as easy recovery and recycling. Among them, the supported noble metal (Ru, Ir and Pd), or transition metal ones (Co, Cu and Ni), show the best catalytic results [12,19,20,21]. Although noble metals show excellent activity and selectivity in the hydrodeoxygenation of LVA to GVL, the industrial use of these catalysts is expensive. To improve the economic efficiency of the processes, it is more expedient to replace them with cost-effective ones [22]. Another drawback is the metal leaching in severe reaction conditions, which limits the applications of noble metals on an industrial scale. The development of an active and cheap catalyst for the preparation of GVL by the hydrodeoxygenation of LVA requires the optimization of the reaction parameters, such as the type of metals and catalyst carriers [23]. Several studies evidence the efficiency of transition metals and their oxides in the catalytic hydrodeoxygenation of LVA to GVL. Almost complete LVA selective conversion has been reported for Ni catalysts supported on various carriers and heterostructured Ni/NiO composites [24,25]. The superior performance of bimetallic transition metal catalysts has also been observed compared to monometallic ones, for example on iron-based bimetallic centers supported on ceramics exceeding 95% GVL conversion at 180 °C and 40 bar H_2_ pressure. The strong synergy of iron oxides with other metals on the catalyst was found to be responsible for the high activity [26]. Bimetallic Ni-Cu/Al_2_O_3_ and Ni-Co/Al_2_O_3_ catalysts have shown 100% conversion also with high GVL selectivity [27]. Similarly, bimetallic Cu-Co/Al_2_O_3_ catalysts showed selectivity over 99% at 250 °C in vapor-phase reaction [28]. The surface morphology, ratio and electronic structure of Brønsted/Lewis acid pairs and Lewis acid/base pairs have been observed to be crucial for the total LVA to GVL conversion, including the hydrogen generation, transfer and hydrogenation steps on transition metal centers [29]. The modification of zeolites with noble or transition metals is a promising approach for the preparation of bifunctional catalysts, with metallic and acidic active species [30]. Faujasite-type zeolites (X or Y) are among the most studied catalytic supports due to the possibility of controlling their catalytic efficiency and selectivity. They can be easily modified with metal or metal oxides, and their supercell structure can host molecules and particles with a large diameter. Metal cations can be incorporated into the structure also by ion exchange and the acidity can be controlled by changing the Si/Al ratio [31]. Metal ions and metal particles in extra-framework positions behaving as Lewis acid centers also have a favorable effect on the catalytic behavior [32]. Finally, environmental safety of zeolites is an important factor, particularly if they are obtained from waste aluminosilicates [33]. The ecological impact of biofuels will be even more significant if we use catalysts that are based on economically profitable and abundantly available waste sources. The approach to obtain efficient and cost-effective catalysts for LVA hydrodeoxygenation to GVL by utilizing solid-phase wastes such as waste incineration fly ash, sewage sludge, and contaminated soil has recently been reported [34]. The published results reveal a 94% conversion efficiency for zeolite-like frameworks modified with NiO [34]. Sulfonic acid-functionalized polystyrene-coated coal fly ash catalyst have been developed for esterification of LVA with n-butanol to alkyl levulinates fuel with 99.6% conversion rate at mild conditions [35]. Solid acid composite catalysts developed based on coal fly ash have shown promising results in biomass conversion processes [36]. Unprocessed coal fly ash, however, is characterized by large non-uniformity in its morphology, structure and composition, and reproducibility can hardly be achieved [37]. In our previous studies, high catalytic activity was found on zeolites obtained from coal fly ash in the oxidation of volatile organic compounds [38]. Coal fly ash zeolites (CFAZ) are self-organized catalytic systems that contain a zeolite phase as a catalytic support with active centers of finely dispersed iron oxide species and framework/extra-framework iron ions incorporated into the zeolite lattice [39]. CFAZ can be easily modified to bimetallic catalysts by post-synthesis impregnation methods with metal salts and their subsequent decomposition to metal oxides, thus increasing their catalytic activity [32]. The processing of coal fly ash into zeolites by double stage alkaline synthesis with ultrasonic homogenization of the reaction mixtures provides several advantages compared to the raw coal ash, such as: tens of times larger specific surface area, mixed micro-mesoporous structure, homogeneous distribution of iron oxide phases, high thermal stability and chemical inertness, reduced leaching of metal particles, etc. [40,41,42]. The favorable surface characteristics of zeolite-like materials in combination with the uniformly incorporated catalytic sites of iron ions and oxide nanoparticles can be useful tools to develop cheap and efficient catalysts. The self-organized catalytic system can be easily modified to obtain bimetallic, highly active catalytic centers for the improvement of catalytic performance.

In the present study, monometallic and bimetallic Ni, Cu and Co functionalized fly ash zeolites were prepared by post-synthesis impregnation and were studied in the hydrodeoxygenation of levulinic acid to γ-valerolactone.

## 2. Results and Discussion

### 2.1. Textural Properties of the Mono- and Bimetallic Zeolite Catalysts

**Figure 1:**
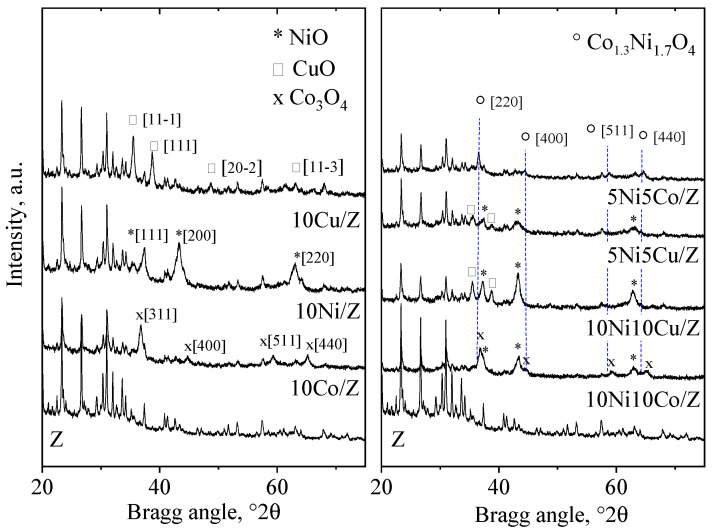
X-ray powder diffraction patterns of the parent, the mono- and bimetallic fly ash zeolites.

The XRD patterns (Figure 1) and the phase analysis of the initial zeolite support show the formation of the following crystalline phases: zeolite 13X (72 wt.%), zeolite A (LTA 3 wt.%), quartz (10 wt.%), dolomite (10 wt.%) and a spinel-type iron oxide, most probably maghemite (5 wt.%) [43,44,45]. The wide amorphous halo at about 25 °2θ is characteristic of a non-crystallized alumino silica phase. Co_3_O_4_, CuO and NiO crystalline phases were registered on the relevant 10 wt.% metal-containing, monocomponent catalysts. The crystallite size of the metal-oxide nanoparticles calculated by the Scherrer equation are presented in Table 1. Cobalt and nickel oxide show higher dispersion on the zeolite support with 17–26 nm crystallite size; however, CuO can be found in more agglomerated form with 35 nm sized nanoparticles. In nickel and copper containing bicomponent formulations both types of metal oxides appear with nickel in higher dispersion [46]. Formation of a Ni_1-x_Cu_x_O solid solution phase with x ˂ 0.2 approximate value is highly probable, because in a NiO/CuO solid solution system incorporation of about 20% of copper was found to be the limit of a separate CuO phase appearance [47,48]. Replacement of Ni^2+^ ions with Cu^2+^ results in some increase in the cubic, F*m*3*m* type unit cell parameter (a_0_) of NiO due to the higher ionic radii of the copper ion (Cu:0.73 Å, Ni: 0.69 Å). However, because of the small difference between the unit cells, the differentiation is difficult with the powder diffraction method, especially with widened reflections due to small crystallite size. Nevertheless, profile fitting with ICDD card No. 01-025-1049 (Ni_0.8_Cu_0.2_O) gives a better result than NiO (01-044-1159). As could be expected, the higher metal content resulted in somewhat bigger crystallite size of the oxides, more enhanced for copper. Separate NiO and Co_3_O_4_ crystalline phases were registered only in the 10Ni10Co/Z sample, together with a nickel cobalt mixed oxide with composition of Co_1.3_Ni_1.7_O_4_ (ICDD card No. 01-040-1191). NiO also showed higher dispersion than cobalt oxide. On the 5Ni5Co/Z catalyst, only the mixed oxide phase could be observed with somewhat lower dispersity. 

**Figure 2:**
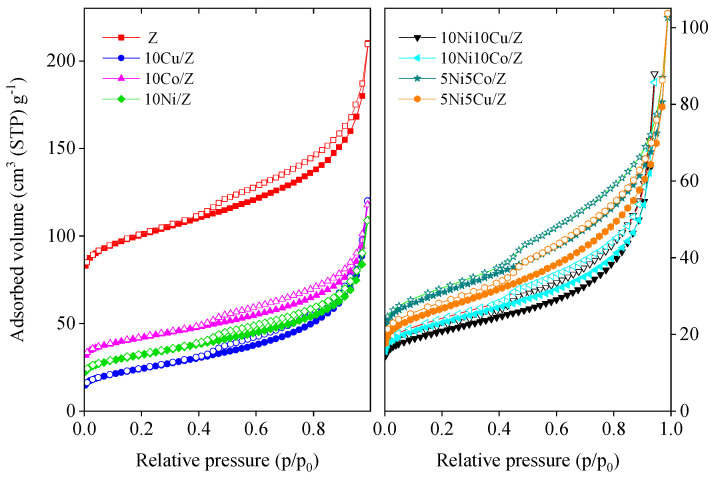
Nitrogen physisorption isotherms of the mono and bimetallic fly ash zeolite materials.

N_2_ adsorption/desorption isotherms of the initial zeolite and its mono- and bimetallic modifications are presented in Figure 2. The isotherms of Z and its modified derivatives are a combination of type II and IV isotherms, typical for microporous zeolites with some mesopore structure [49]. The isotherms show H3 type of hysteresis loops, characteristic of slit-like pores with wide distribution of pore diameter. The calculated textural parameters are summarized in Table 2. The modification of the initial fly ash zeolite with Co, Cu and/or Ni leads to a significant decrease in the surface area and micropore volume, indicating the pore blocking of the zeolite. Most probably metal ions were incorporated into the zeolite lattice in cationic positions. Mesopore volume also decreased, showing that nanosized metal oxide deposits also occupied the pore system. The extent of decrease is in line with the chemical nature of incorporating cations. The monovalent copper can more easily be exchanged into the zeolite lattice compared to cobalt. Therefore, the 10Cu/Z sample shows much lower textural characteristics than 10Co/Z. Bimetallic catalysts with 20 wt.% metal content have a lower specific surface area and pore volume due to the lower amount of adsorbing zeolite phase. 

To support the above ideas the nitrogen physisorption isotherms of some 600 °C reduced samples were also measured. A significant increase in specific surface area and micropore volume was experienced (Table 2), as evidence that upon reduction the metal ions and particles are migrated to the surface and made the pores accessible to adsorbing gas again. The zeolite structure also remained intact after reduction, as proven by the XRD results.

**Figure 3:**
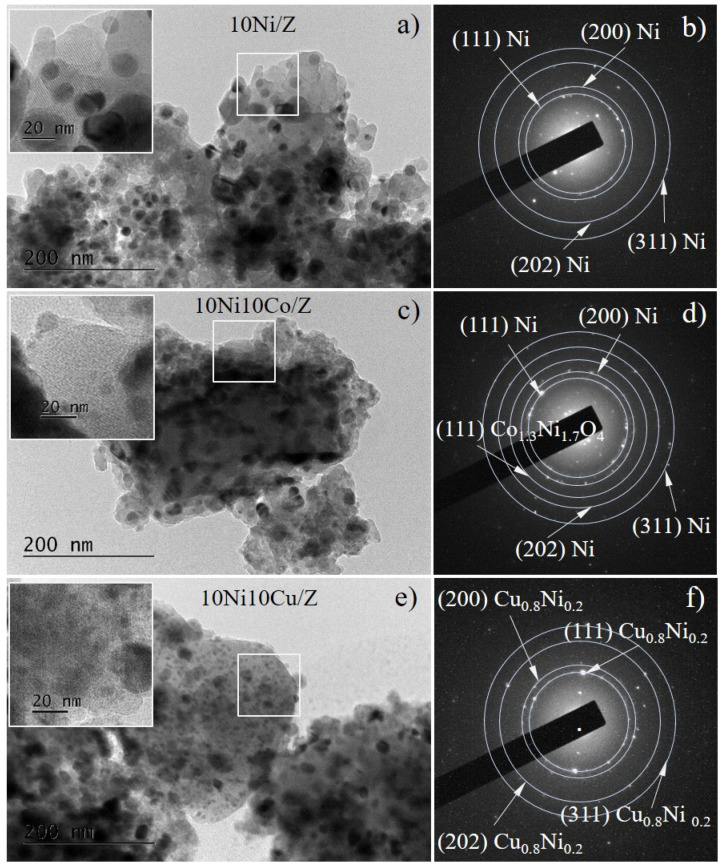
Bright Field TEM images (**a**,**c**,**e**) and the corresponding SAED patterns (**b**,**d**,**f**) of 10Ni/Z (**a**,**b**), 10Ni10Co/Z (**c**,**d**) and 10Ni10Cu/Z catalysts after reduction.

In Figure 3a,c,e the bright-field TEM images of the reduced catalysts 10Ni/Z, 10Ni10Co/Z and 10Ni10Cu/Z are presented. In all samples, the morphology of the spherical-like metal nanoparticles dispersed on the zeolite support is illustrated. The insets show magnified images of the marked squared areas of the corresponding micrographs, revealing the zeolite microstructure. Selected area electron diffraction patterns permit to identify the samples phase composition. A metallic Ni phase (COD Entry # 96-901-3028) was registered in three catalysts, which was also detected by the XRD and XPS analysis of these samples. The presence of a mixed compound Co_1.3_Ni_1.7_O_4_ and Cu_0.8_Ni_0.2_ alloy was found in the bimetallic 10Ni10Co/Z and 10Ni10Cu/Z catalyst, respectively, in good agreement with X-ray diffraction data.

### 2.2. Redox and Surface Analytical Characteristics of Mono- and Bimetallic Catalysts

**Figure 4:**
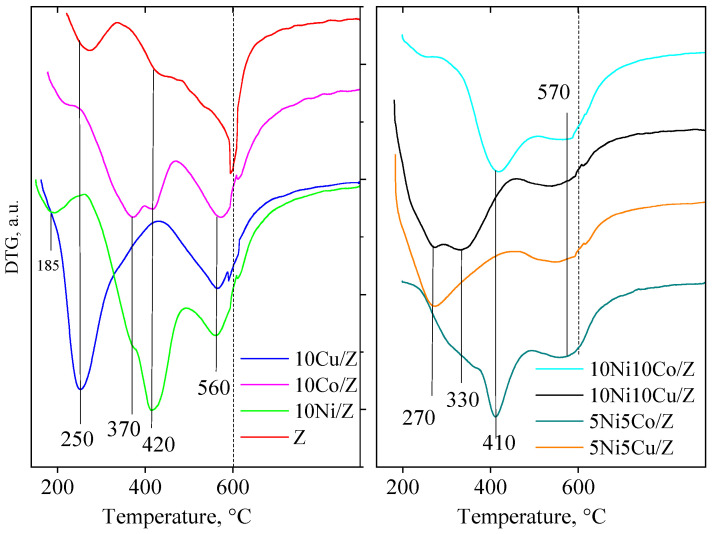
TPR-TG profiles of the studied catalysts.

The redox properties of the mono- and bimetallic fly ash zeolite materials were studied by TPR experiments (Figure 4). The parent zeolite support contains 7 wt.% of iron by ICP analysis. As is typical for highly dispersed iron oxides, the reduction takes place at about 400–450 °C, but iron can occupy also hardly reducible cationic positions. Accordingly, the TPR curve of the fly ash zeolite shows peaks between 350 and 600 °C. The reduction rate is 100%, so most of the iron is reducible. The reduction of cobalt oxide on the fly ash zeolite is characterized by three steps, at 325, 370 and 570 °C, which suggests that a part of the cobalt oxide is finely dispersed on the support, but a similar part is hardly reducible. 

The latter one can be identified with cobalt ions in cationic position of the zeolite or by formation of cobalt silicate. Monometallic Ni-material can be reduced at a similar temperature interval as the cobalt sample, showing three reduction peaks at around 325, 420 and 570 °C, also indicating the formation of finely dispersed nickel oxide particles, and cationic nickel in the zeolite lattice.

In contrast, the copper zeolite can be reduced in one intense step at a much lower temperature, at 250 °C, and in a higher, but less intensive one over 560 °C. The identification is similar to the preceding ones, finely dispersed copper oxide on the external surface and copper ions in the zeolite lattice, respectively.

The modification with two metals resulted in significant changes in the reduction behavior of copper-containing samples. The presence of copper enhanced the reducibility of nickel, most probably by the formation of an intermetallic phase with copper [10]. Ni and cobalt containing catalysts can be reduced in the same temperature range as the individual components, i.e., over 400 °C.

The extent of metal oxide reduction is calculated based on the amount of hydrogen consumption during reduction and the results are presented in Table 1. Total reduction was calculated for all the samples except cobalt containing bimodal catalysts. This may be related to the formation of a hard-to-reduce cobalt silicate due to the high metal concentration in the catalyst.

**Figure 5:**
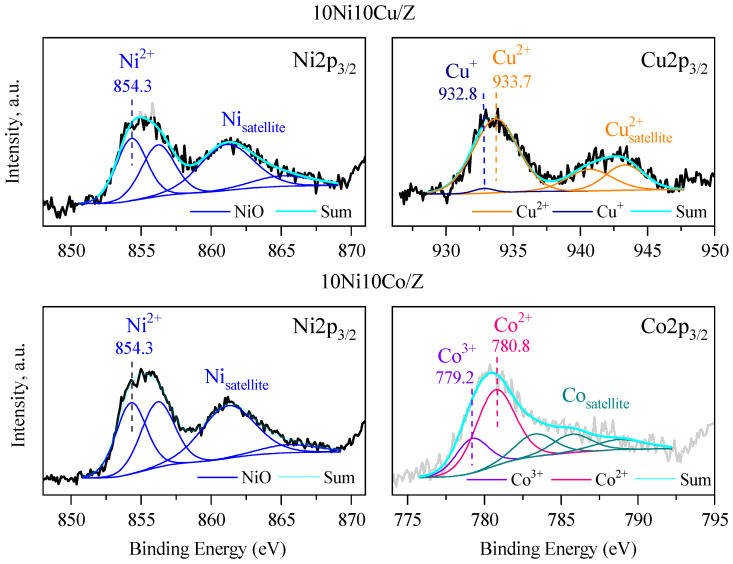
XPS spectra of the 10Ni10Co/Z and 10Ni10Cu/Z catalysts.

The surface composition of bimetallic samples with 20% metal content was analyzed via X-ray photoelectron spectroscopic (XPS) method. The spectra of 10Ni10Co/Z, and 10Ni10Cu/Z catalysts are presented in Figure 5. The calculated compositions are summarized in Table 3. The Ni 2p_3/2_ peak with binding energy of 853.7 eV is characteristic of Ni^2+^ in NiO [50], and its shift to higher binding energy (854.3 eV) can indicate the interaction with Si or Cu/Co. The peaks in the Cu 2p_3/2_ spectrum at 933.4 eV and the presence of the characteristic satellites support the XRD data, showing the presence of CuO phase [51]. In the Co2p_3/2_ spectrum, the peaks at 779.2 (Co^3+^) and 780.8 (Co^2+^) eV support the formation of a spinel Co_3_O_4_ phase (Figure 4). The surface chemical composition mirrors the crystalline phases of the catalysts with the presence of Fe, Ca and Mg, whereas Na, Si and Al are the main constituents of zeolite support and amorphous silica alumina phase.

The amount of catalytically active Ni, Cu and Co elements differs from that of the bulk value, the nickel is overrepresented 2–3 times on the surface. This can be explained by the formation of nickel-rich mixed oxides and the interaction of copper and cobalt with the silica matrix. The presence of bigger copper and cobalt oxide nanoparticles can be another reason, in accordance with the XRD results.

### 2.3. Catalytic Behavior of Mono- and Bimetallic Catalysts in Hydrodeoxygenation of LVA to GVL

The catalytic activity of the mono-metallic and bimetallic zeolites was studied in hydrodeoxygenation of levulinic acid to γ-valerolactone at 150 °C reaction temperature, and the results are presented in Table 4.

**Scheme 1:**
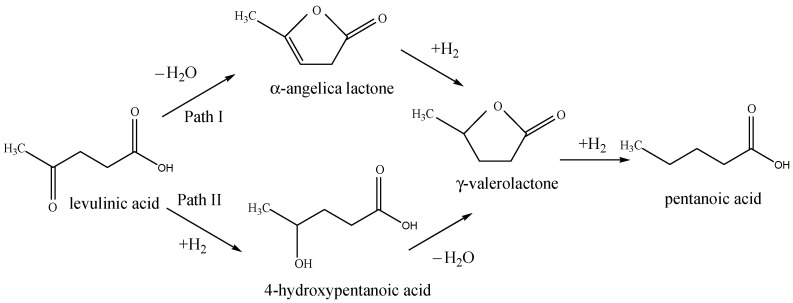
Reaction mechanisms of LVA hydrodeoxygenation to GVL.

The main detected products in the reaction are γ-valerolactone (GVL) and 4-hydroxy pentanoic acid (HPA). Low catalytic activity was registered for the cobalt and copper containing monometallic catalysts. However, 10Ni/Z showed almost 10 times higher activity than the former ones. A significant increase in the catalytic activity of bimetallic catalysts was observed, especially at increased metal content, with about 100% conversion. The γ-valerolactone yield exceeded that of 4-hydroxy pentanoic acid in each case. According to Scheme 1, the formation of GVL is reported via two possible pathways. When the dehydrogenation reaction step is more decisive, Path I proceeds by the formation of α-angelica lactone, and it is rather characteristic of the hydrodeoxygenation reaction in the vapor phase [10]. In the liquid phase reaction, 4-hydroxypentanoic acid is experienced to be the typical intermediate in the consecutive reaction, due to lower reaction temperature, thermodynamically favoring the hydrogenation as a first reaction step. Based on the obtained reaction products we suppose that Path II is dominating the reaction (Scheme 1) without a further hydrogenation step to pentanoic acid. The synergistic effect of bimetallic catalysts is well-marked compared to monometallic ones. One explanation can be the formation of mixed oxide phases due to the improved reducibility of the active components. However, the amount of metals also plays a crucial role. With higher metal content, the formation of active metallic phase by reduction is more pronounced, due to lower interaction with the support, and the formation of highly dispersed metallic phases. When the stability tests were performed with recycled samples (Table 4), the monometallic catalysts showed a significant decrease in activity. The effect was especially pronounced on the 10Ni/Z catalyst, with a decrease from 35 wt.% to ~15% of LA conversion. However bimetallic 10Ni10Co/Z and 10Ni19Cu/Z catalysts exhibited higher stability, with less than 10% activity loss for the second run (from 99.5 wt.% to 92.7 wt.% and 100 wt. % to 95.3 wt.%, respectively). By longer reaction time experiments (8 h) some further activity change of 10Ni10Co/Z catalyst from 99.5% to 87 wt. %. could be observed. It seems that the high initial hydrogenation activity of 10Ni/Z sample is in connection with the favorable distribution of nickel on the surface of zeolite support, providing much higher dispersion compared to copper and cobalt containing samples. Copper oxide on the zeolite support can more easily be reduced; however, it is more easily agglomerated. Cobalt oxide is hardly reducible, and cobalt is prone to form copper silicate with the support, therefore only a small part of it can be in metallic state during the reaction. By bimetallic catalysts, formation of mixed oxides was experienced, i.e., nickel is diluted and stabilized in an intermetallic phase by reduction, preserving its high dispersion. 

The comparison of catalytic performance of the fly ash-based catalysts with published data are presented in Table 5.

In water solvent condition, Ni/Al_2_O_3_ was reported to achieve 100% LA conversion with 82% GVL selectivity (200 °C, 50 bar H_2_) [52], while bimetallic Ni-Cu/Al_2_O_3_ [59] reported 100% LA conversion with 96% GVL selectivity at 250 °C at 65 bar H_2_ for 2 h. Significant LA conversion and selectivity to GVL is reached at 270 °C and 0.05 g on Ni/TiO_2_ catalyst in the vapor phase. Cu/Ni/Mg/Al (hydrotalcite-derived) catalyst [55] showed total LA conversion and selectivity to GVL at 200 °C and 30 bars H_2_. Our bimetallic catalysts (10Ni10Co/Z and 10Ni10Cu/Z) also showed total LA conversion and selectivity to GVL at lower reaction temperature (150 °C) and lower amount of catalyst (0.2 g). We assume that the presence of Fe in the zeolite support positively influences the metal nanoparticles dispersion. 

**Figure 6:**
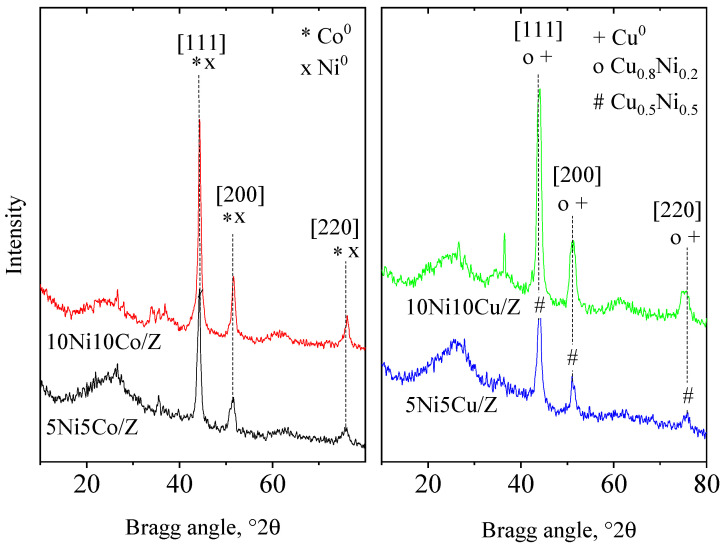
XRD patterns of spent bimetallic catalysts.

Investigation of the spent catalysts supported the above observations and revealed some other important characteristics of the catalytic system. The XRD patterns of the four most active catalysts are shown in Figure 6. The absence of crystalline zeolite phase is evident, and formation of only metallic phases is experienced. Nitrogen physisorption data (Table 6) support the lack of a zeolite phase, because micropores cannot be detected in the samples. However, the surface area of the amorphous silica alumina is high enough to serve as a catalyst support, and the increased pore volume values indicate the reconstruction of the pore system. The phenomena of amorphization can be explained by using water as a solvent, promoting the hydrothermal decomposition of the zeolite. Investigating the phase composition of metallic nanoparticles produced by reduction, the formation of intermetallic phases is highly probable. In Ni/Co composition, the substitution of ions cannot be proved by the XRD method, because of the similarities of the ion size and cubic unit cell of the two metals. The formation of NiCu alloys was identified on both Ni/Cu catalysts, but with different compositions. In the 5Ni5Cu/Z sample, the ratio of the metals was equal, based on the calculation of unit cell size of the cubic system (a_0_ = 3.56 Å), according to Vegard’s law, presented by the works of Smirnov et al. [60]. In the 10Ni10Cu/Z sample, the presence of copper-rich phases was detected. The dispersion of metals was higher than that of metal oxides in the parent catalysts.

## 3. Experimental Part

### 3.1. Materials

The raw material for the preparation of the catalysts is a coal fly ash collected from the electrostatic precipitators of one of the largest combustion plants in Bulgaria, TPP ‘AES Galabovo’, burning lignite coal. CFA has been studied in our previous publications with respect to chemical and phase composition and morphology [43,44]. It is a high silica CFA grade F, according to ASTM C618-23 standard [61], containing about 74 wt.% SiO_2_ + Al_2_O_3_, of which 50 wt. % is SiO_2_. CFA has a low CaO content of up to 4.5 wt.% and includes a larger amount of iron oxide phases, expressed as Fe_2_O_3_, approx. 13 wt.%. Due to the characteristics of the steam generator, CFA is highly amorphized, but can contain some crystalline phases such as quartz, mullite, magnetite and portlandite [44,45].

### 3.2. Synthesis of Coal Fly Ash Zeolite

The initial zeolite from coal ash, denoted as **Z**, was synthesized by ultrasound-assisted two-step synthesis comprising of hydrothermal activation with pre-alkaline fusion. Coal fly ash and sodium hydroxide used as an alkaline activator were mixed in an appropriate ratio and calcined at 550 °C for 1 h. The cooled batch was crushed, mixed with distilled water to a medium alkalinity of 2.3 mol/l and homogenized by ultrasonic treatment for 15 min. The resulting suspension was aged for 8 h and subjected to hydrothermal activation at 90 °C for 4 h. The obtained powdery product was removed by filtration, washed with distilled water until neutral pH and dried at 105 °C. This well-established and optimized laboratory procedure for coal fly ash alkaline conversion yields high-quality zeolite X as a single crystalline phase with surface characteristics favorable for catalytic applications [40,43].

### 3.3. Impregnation of Coal Fly Ash Zeolite with Ni, Co and Cu

An impregnation technique with nickel, cobalt and copper salts was applied for loading of 10 wt.% metals. The zeolite support was dehydrated at 160 °C for 2 h before the impregnation procedure.

The monometallic fly ash zeolites were prepared by the following procedures: 10 wt.% Ni: 550 mg Ni(NO_3_)_2_·6H_2_O was dissolved in 1 mL distilled water and was added to 1 g zeolite support by stirring until evaporation of the solvent. Then, the sample was dried at 80 °C for 18 h and calcined at 450 °C for 3 h with a rate of 1 °C/min. The sample was denoted as 10Ni/Z; 10 wt.% Co: 548.2 mg Co(NO_3_)_2_·6H_2_O was dissolved in 1 mL distilled water and was added to 1 g AES0 support by stirring until evaporation of the solvent. Then, the sample was dried at 80 °C for 18 h and calcined at 450 °C for 3 h with a rate of 1 °C/min. The sample was denoted as 10Co/Z; 10 wt.% Cu: 516.4 mg Cu(NO_3_)_2_·6H_2_O was dissolved in 1 mL distilled water and was added to 1 g zeolite support by stirring until evaporation of the solvent. Then, the sample was dried at 80 °C for 18 h and calcined at 450 °C for 3 h with a rate of 1 °C/min. The sample was denoted as 10Cu/Z.

Bimetallic, 5 or 10 wt.% Ni- and Co-modified fly ash zeolite was prepared by the following procedures: 275/550 mg Ni(NO_3_)_2_·6H_2_O and 274.1/548.2 mg Co(NO_3_)_2_·6H_2_O dissolved in 1 mL distilled water was added to 1 g fly ash zeolite sample and then was dried at 80 °C for 18 h. The precursor salts were decomposed in air at 450 °C with a rate of 1 °C/min for 3 h. The samples were denoted as 5Ni5Co/Z and 10Ni10Co/Z, respectively.

Bimetallic, 5 or 10 wt.% Ni- and Cu-modified fly ash zeolite was prepared by the following procedures: 275/550 mg Ni(NO_3_)_2_·6H_2_O and 258.2/516 mg Cu(NO_3_)_2_·6H_2_O dissolved in 1 mL distilled water was added to 1 g zeolite and then was dried at 80 °C for 18 h. The precursor salts were decomposed in air at 450 °C with a rate of 1 °C/min for 3 h. The samples were denoted as 5Ni5Cu/Z and 10Ni10Cu/Z, respectively.

### 3.4. Characterization

X-ray powder diffraction patterns were recorded by a Philips X’Pert type (Bruker AXS Advanced X-ray Solutions GmbH, Karlsruhe, Germany) diffractometer applying monochromatized CuKα radiation (40 kV, 35 mA). Patterns were collected between 3 and 75 °2θ with 0.02° step size for 4 s. Crystallite size of the metal oxides was determined by the Sherrer equation evaluating the FWMH values of the oxide phases with full profile fitting method. The list of ICDD cards used for identification of metal oxide and metal phases is the following: CuO: 00-048-1548; NiO: 00-078-0643; Co_3_O_4_: 00-009-0418; Co_1.29_Ni_1.71_O_4_: 00-040-1191; Ni_0.8_Cu_0.2_O: 01-078-0647; Ni^0^: 00-045-1027; Cu^0^: 00-004-0836; Co^0^: 00-015-0806; Cu_0.81_Ni_0.19_: 00-047-1406; Ni_x_Cu_1-x_: 01-066-0202.

Specific surface area and pore volume of the samples was determined from N_2_ physisorption isotherms collected at −196 °C using AUTOSORB iQ-C-MP-AG-AG (Quantachrome Instruments, Anton Paar brand, Boynton Beach, FL, USA). Samples were pretreated at 350 °C in vacuum before nitrogen adsorption. Total pore volume was determined according to the Gurvich rule at relative pressure of 0.9.

The temperature-programmed reduction–thermogravimetric analysis (TPR-TGA) investigations were performed by a STA449F5 Jupiter type instrument of NETZSCH Gerätebau GmbH (Selb, Germany). In a typical measurement 20 mg of sample was placed in a microbalance crucible and heated in a flow of 5 vol. % H_2_ in Ar (100 cm^3^/min) up to 600 °C at a rate of 5 °C/min and a final hold-up of 1 h. Prior to the TPR experiments the samples were treated in situ at 500 °C in air flow (10 °C/min) for 1 h.

The XPS measurements were carried out on an AXIS Supra electron spectrometer (Kratos Analytical Ltd., Manchester, UK) using AlK_α_ radiation with photon energy of 1486.6 eV. The energy calibration was performed by normalizing the C1s line of adsorbed adventitious hydrocarbons to 284.8 eV. The binding energies (BE) were determined with accuracy of ±0.1 eV using the commercial data-processing software ESCApe^TM^ version 1.2.0.1325 from Kratos Analytical Ltd. The concentrations of the different chemical elements (in atomic %) were calculated by normalizing the areas of the photoelectron peaks to their relative sensitivity factors. The deconvolution of the peaks has been performed by using the commercial data-processing software ESCApe^TM^ of Kratos Analytical Ltd.

The morphology and the phase composition of the spent catalysts was followed by means of Transmission Electron Microscopy. Transmission electron microscope JEOL JEM 2100 (JEOL Ltd., Tokyo Japan) was used for this purpose. Three regimes of the microscope were applied to accumulate the information: conventional Bright Field TEM mode (BF TEM), Selected Area Electron Diffraction (SAED) and High-Resolution mode (HR TEM). A preliminary preparation procedure of the samples was applied—the powders were suspended in analytically pure ethanol, ultra-sonicated for 3 min and dropped and dried on standard TEM Cu grid, covered with carbon film.

### 3.5. Catalytic Experiments

Prior to the catalytic tests, the samples were pretreated for 1 h in Ar/H_2_ flow at 500 °C, with 1 °C/min rate. In a typical experiment, the reactor was charged with 1 g LVA (Sigma-Aldrich, 98%, Burlington, MA, USA), 20 mL H_2_O and 0.2 g powder catalyst while the LVA/H_2_O weight ratio was maintained at 1:10.

Levulinic acid hydrodeoxygenation was studied at atmospheric pressure using a 100 mL stainless steel batch reactor with hydrogen as a carrier gas (30 mL/min). The reactor was heated under stirring at 700 rpm at a reaction temperature of 150 °C for 4 h or 8 h. The thermocouple was positioned in the reaction mixture for the accurate measurement of the reaction temperature. Samples were taken every hour from the reaction mixture starting from 1 h reaction time and analyzed using HP-GC with a Shimadzu column, cat. Nº221-75940-30 Phase: SH-Rxi; -5MS; Size L30m. Levulinic acid conversion was calculated as X_LA_ = (C°_LA_ − C^t^_LA_)/C°_LA_ × 100 where C°_LA_ and C^t^_LA_ are the amount of initial and amount of LA at ***t*** reaction time. The yield of GVL was calculated as Y_GVL_ = C_GVL_/C°_LA_ × 100 and yield of 4-HPA was calculated as Y_4-HPA_ = C_4-HPA_/C°_LA_ × 100.

## 4. Conclusions

Monometallic (Ni, Cu, Co) and bimetallic (Ni-Co, Ni-Cu) catalysts supported on fly ash zeolite were prepared by post-synthesis impregnation method. Formation of metal oxides and mixed metal oxides was detected on the support. The blocking of the micropore and mesopore system by metal ions and highly dispersed metal oxides was also an accompanying phenomenon. By reducing the catalysts, the pore system became permeable again. TPR experiments proved the favorable effect of bimetallic systems on improved reducibility with Ni/Cu compositions. An XPS study revealed the enrichment of nickel on the surface of bimetallic compositions with high metal content. In accordance with physico-chemical characterization results, high catalytic activity was detected for levulinic acid hydrodeoxygenation to γ-valerolactone on bimetallic catalysts with 20 wt.% metal content. Besides the total conversion, the yield to GVL was also high, over 80%. Investigation of the spent catalysts showed the collapse of the zeolite structure and reorganization of the pore system, creating a high-surface-area amorphous silica-alumina supported Ni/Co, Ni/Cu catalytic system.

## Figures and Tables

**Table 1:** Crystallite size of the metal oxides and reducibility of the studied catalysts.

Sample Name	Crystalline Phase	Crystallite Size, (nm)	Reducibility, %
10 Ni/Z	NiO	17	100
10 Co/Z	Co_3_O_4_	26	79
10Cu/Z	CuO	35	100
10Ni10Co/Z	NiO	17	82
	Co_1.3_Ni_1.7_O_4_	26	
	Co_3_O_4_	26	
10Ni10Cu/Z	NiO/Ni_0.8_Cu_0.2_O	17	100
	CuO	35	
5Ni5Co/Z	Co_1.3_Ni_1.7_O_4_	35	100
5Ni5Cu/Z	NiO/Ni_0.8_Cu_0.2_O	13	100
	CuO	21	

**Table 2:** Textural properties of the studied catalysts.

Samples	S_BET_ (m^2^/g)	Micropore Volume (cm^3^/g)	Total pore Volume (cm^3^/g)
10Ni/Z	115	0.019	0.169
10 Co/Z	152	0.034	0.183
10Cu/Z	86	0.006	0.186
10Ni10Co/Z	82	0.015	0.133
10Ni10Cu/Z	75	0.013	0.128
5Ni5Co/Z	111	0.020	0.159
5Ni5Cu/Z	96	0.015	0.160
Z	380	0.107	0.326
Samples reduced at 600 °C			
10Ni10Co/Z	181	0.047	0.130
5Ni5Co/Z	228	0.061	0.152
10Ni/Z	240	0.066	0.154

**Table 3:** XPS data of 10Ni10Co/Z and 10Ni10Cu/Z catalysts.

Composition, at. %	Cu	Ni	Co	Si	Al	O	Fe	Na	Ca	Mg
10Ni10Cu/Z	3.2	7.3	-	10.4	4.5	61.4	3.9	6.5	1.4	1.4
10Ni10Co/Z	-	11.4	3.9	9.1	3.5	60.3	5.0	4.0	1.3	1.5

**Table 4:** Catalytic data for the hydrodeoxygenation of levulinic acid on the studied catalysts.

Catalysts	Conversion, %	HPA Yield, %	GVL Yield, %
10Ni/Z	35.0/14.9 *	8.0/1.3 *	27.0/13.6 *
10Co/Z	6.5/2.7 *	1.7/0.8 *	4.8/1.9 *
10Cu/Z	8.3/5.9 *	2.0/1.3 *	6.3/4.6 *
5Ni5Co/Z	51.0	7.0	44.0
5Ni5Cu/Z	63.0	10.0	53.0
10Ni10Co/Z	99.5/92.7 *	14.8/13.8 *	84.7/78.9 *
10Ni10Cu/Z	100.0/95.3 *	15.0/14.5 *	85.0/80.8 *

* catalytic data with recycled catalysts.

**Table 5:** LA conversion to GVL on different catalysts (water is applied as solvent).

Catalysts	LA Conversion (%)	GVL Yield (%)	H Donor	Temp, Time	LA Loading	Catalyst Quantity	Ref.
Ni/Al_2_O_3_ (5 wt%Ni)	100	82	50 bar H_2_	200 °C	5 g/h	0.5 g	[52]
Ni/SiO_2_	15.7	90.9	H_2_ 20 mL/min	270 °C Vapor phase	10 wt. % in water	0.05 g	[53]
Ni/TiO_2_ (10 wt. % Ni)	99.9	99.1	H_2_ 20 mL/min	270 °C Vapor phase	10 wt. % in water	0.05 g	[54]
Cu/Ni/Mg/Al (hydrotalcite-derived)	100	100	30 bar H_2_	200 °C 6 h	1 g, 2.5 wt.%	0.161 g	[55]
Cu/ZrO_2_	100	100	35 bar H_2_	200 °C 5 h	5 mL	0.5 g	[56]
Cu/SiO_2_	48	38.4	Formic acid	250 °C, 10 h Vapor phase	LA/FA 28:22 wt.%	0.5 g	[57]
Cu/ZSM-5	100	99	50 bar H_2_	200 °C 6 h	0.25 g	0.1 g	[58]
Ni-Cu/Al_2_O_3_	100	96	65 bar H_2_	250 °C 2 h	10 mL	1 g	[59]

**Table 6:** Textural parameters of spent catalysts and the crystallite size of reduced metals.

Samples	S_BET_ (m^2^/g)	Total Pore Volume (cm^3^/g)	Crystalline Phases	Crystallite Size, nm
10Ni10Co/Z spent	275	0.462	Ni^0^/Co^0^	21
10Ni10Cu/Z spent	271	0.422	Cu^0^, Cu_0.81_Ni_0.19_	13/13
5Ni5Co/Z spent	256	0.379	Ni^0^/Co^0^	19
5Ni5Cu/Z spent	279	0.400	Ni_x_Cu_1-x_ (x~0.5)	13

## Data Availability

Data are contained within the article.
