# Correction: Popova et al. Ni-Cu and Ni-Co-Modified Fly Ash Zeolite Catalysts for Hydrodeoxygenation of Levulinic Acid to γ-Valerolactone. *Molecules* 2024, *29*, 99

**DOI:** 10.3390/molecules29112648

**Published:** 2024-06-04

**Authors:** Margarita Popova, Momtchil Dimitrov, Silviya Boycheva, Ivan Dimitrov, Filip Ublekov, Neli Koseva, Genoveva Atanasova, Daniela Karashanova, Ágnes Szegedi

**Affiliations:** 1 Institute of Organic Chemistry with Centre of Phytochemistry, Bulgarian Academy of Sciences, 1113 Sofia, Bulgaria; momtchil.dimitrov@orgchm.bas.bg (M.D.); ivan.dimitrov@orgchm.bas.bg (I.D.); 2 Department of Thermal and Nuclear Power Engineering, Technical University, 1756 Sofia, Bulgaria; sboycheva@tu-sofia.bg; 3 Institute of Polymers, Bulgarian Academy of Sciences, 1113 Sofia, Bulgaria; fublekov@polymer.bas.bg (F.U.); koseva@polymer.bas.bg (N.K.); 4 Institute of General and Inorganic Chemistry, Bulgarian Academy of Sciences, 1113 Sofia, Bulgaria; genoveva@svr.igic.bas.bg; 5 Institute of Optical Materials and Technologies, Bulgarian Academy of Sciences, 1113 Sofia, Bulgaria; dkarashanova@yahoo.com; 6 HUN-REN, Research Centre for Natural Sciences, Institute of Materials and Environmental Chemistry, Magyar Tudósok krt. 2., 1117 Budapest, Hungary

**Error in Figure**

In the original publication [1], there was a mistake in “Scheme 1. Reaction mechanisms of LVA hydrodeoxygenation to GVL” as published. There is a technical mistake in the formula of γ-valerolactone.

Please see the corrected version below of Scheme 1.

**Text Correction**

There was an error included in the original publication. There was a mistake in the stated solvent.

A correction has been made to 3. Experimental Part, 3.3. Impregnation of Coal Fly Ash Zeolite with Ni, Co and Cu, paragraphs 2, 3 and 4.

The monometallic fly ash zeolites were prepared by the following procedures: 10 wt.% Ni: 550 mg Ni(NO_3_)_2_·6H_2_O was dissolved in 1 mL distilled water and was added to 1 g zeolite support by stirring until evaporation of the solvent. Then, the sample was dried at 80 °C for 18 h and calcined at 450 °C for 3 h with a rate of 1 °C/min. The sample was denoted as 10Ni/Z; 10 wt.% Co: 548.2 mg Co(NO_3_)_2_·6H_2_O was dissolved in 1 mL distilled water and was added to 1 g AES0 support by stirring until evaporation of the solvent. Then, the sample was dried at 80 °C for 18 h and calcined at 450 °C for 3 h with a rate of 1 °C/min. The sample was denoted as 10Co/Z; 10 wt.% Cu: 516.4 mg Cu(NO_3_)_2_·6H_2_O was dissolved in 1 mL distilled water and was added to 1 g zeolite support by stirring until evaporation of the solvent. Then, the sample was dried at 80 °C for 18 h and calcined at 450 °C for 3 h with a rate of 1 °C/min. The sample was denoted as 10Cu/Z.

Bimetallic, 5 or 10 wt.% Ni- and Co-modified fly ash zeolite was prepared by the following procedures: 275/550 mg Ni(NO_3_)_2_·6H_2_O and 274.1/548.2 mg Co(NO_3_)_2_·6H_2_O dissolved in 1 mL distilled water was added to 1 g fly ash zeolite sample and then was dried at 80 °C for 18 h. The precursor salts were decomposed in air at 450 °C with a rate of 1 °C/min for 3 h. The samples were denoted as 5Ni5Co/Z and 10Ni10Co/Z, respectively.

Bimetallic, 5 or 10 wt.% Ni- and Cu-modified fly ash zeolite was prepared by the following procedures: 275/550 mg Ni(NO_3_)_2_·6H_2_O and 258.2/516 mg Cu(NO_3_)_2_·6H_2_O dissolved in 1 mL distilled water was added to 1 g zeolite and then was dried at 80 °C for 18 h. The precursor salts were decomposed in air at 450 °C with a rate of 1 °C/min for 3 h. The samples were denoted as 5Ni5Cu/Z and 10Ni10Cu/Z, respectively.

The authors state that the scientific conclusions are unaffected. This correction was approved by the Academic Editor. The original publication has also been updated.

## Figures and Tables

**Scheme 1:**
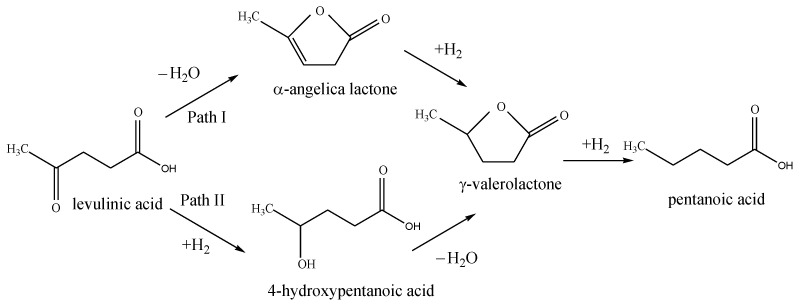
Reaction mechanisms of LVA hydrodeoxygenation to GVL.
